# *IL17A* and *IL17F* genes polymorphisms are associated with histopathological changes in transplanted kidney

**DOI:** 10.1186/s12882-019-1308-z

**Published:** 2019-04-08

**Authors:** Leszek Domanski, Karolina Kłoda, Maciej Patrzyk, Magda Wisniewska, Krzysztof Safranow, Jerzy Sienko, Tadeusz Sulikowski, Marzena Staniszewska, Andrzej Pawlik

**Affiliations:** 1 Department of Nephrology, Transplantology and Internal Medicine, Pomeranian Medical University in Szczecin, Szczecin, Poland; 2 Department of Surgery, University Medical Center Greifswald, Greifswald, Germany; 3 Department of Biochemistry and Medical Chemistry, Pomeranian Medical University in Szczecin, Szczecin, Poland; 4 Department of Surgery, Pomeranian Medical University in Szczecin, Szczecin, Poland; 5 Department of Physiology, Pomeranian Medical University in Szczecin, Powstancow Wlkp. 72, 70-111 Szczecin, Poland

**Keywords:** Cytokines, Histopathology, IL-17, Kidney allograft, Polymorphism

## Abstract

**Background:**

Interleukin 17 is a proinflammatory cytokine involved in immune response after allograft transplantation. IL-17 family of proinflammatory cytokines includes *IL-17A* and *IL-17F*. Previous studies have demonstrated that the rs2275913 *IL17A* and the rs11465553 *IL17F* gene polymorphism are associated with kidney allograft function. Because of the association between these polymorphisms and post-transplant immune response, we assume that these single nucleotide polymorphisms may affect morphological structure of transplanted kidney.

The aim of this study was to examine the association of rs2275913 *IL17A* and rs2397084, rs11465553 and rs763780 *IL17F* gene polymorphisms with histopathological changes in transplanted kidney biopsies such as: glomerulitis, tubulitis, arteritis, cell infilitration and fibrosis.

**Methods:**

The study enrolled 82 patients after renal graft transplantation in whom a kidney biopsy was performed because of impaired graft function. The rs2397084 T > C (Glu126Gly), rs11465553 G > A (Val155Ile) and rs763780 T > C (His167Arg) polymorphisms within the *IL17F* gene and the rs2275913 A > G (− 197 A > G) polymorphism within the *IL17A* gene promoter were genotyped using TaqMan genotyping assays on a 7500 FAST Real-Time PCR System (Applied Biosystems, USA).

**Results:**

There was a significant association between the rs2275913 *IL17A* gene polymorphism and the grade of tubulitis, which was more severe among patients with the A allele, compared to recipients with the GG genotype (GG vs. AG + AA, *P* = 0.02), and with the grade of arteriolar hyaline thickening and mesangial matrix increase, which were more severe among patients with the G allele compared to recipients with the AA genotype (AA vs. AG + GG, *P* = 0.01 and *P* = 0.04, respectively). Tubular atrophy and interstitial fibrosis were more severe among individuals with the C allele at the rs763780 *IL17F* gene polymorphism (TT vs. TC, *P* = 0.09 and *P* = 0.017, respectively). However, it should be taken into account that the statistical significance was achieved without correction for multiple testing, and no significant association would remain significant after such correction.

**Conclusions:**

The results of this study may suggest a possible association between the rs2275913 *IL17A* and rs2275913 *IL17A* gene polymorphisms and some histopathological changes in transplanted kidney biopsies.

**Electronic supplementary material:**

The online version of this article (10.1186/s12882-019-1308-z) contains supplementary material, which is available to authorized users.

## Background

Interleukin 17 (IL-17) is a member of the IL-17 family of cytokines, which includes among others IL-17A and IL-17F. [1]. The complex structure of IL-17 and its pleiotropic function are still being investigated [2]. It was suggested that this cytokine forms a link between the innate and adaptive immune response [3]. Induction with IL-23 leads to enhanced IL-17 secretion by Th17, CD8^+^ T cells and γδ T cells [4]. The biological activity of IL-17 is associated with proinflammatory actions in allergies, autoimmune diseases and cancer [3], moreover, it is also an important mediator in bacterial infections. However, recent studies revealed that IL-17 may also possess inhibitory functions and suppress the inflammatory response [3, 5, 6]. Numerous reports have confirmed that cytokine synthesis has a genetic background [7]. Genes encoding IL-17A and IL-17F are located on chromosome 6 (6p12) [8], and polymorphisms in these genes were studied in various autoimmune diseases [4, 9]. Studies regarding bone marrow and solid organ transplantation have highlighted the importance of *IL17A* and *IL17F* gene polymorphisms in the pathogenesis of rejection; however, the nature of the posttransplant immune response is complex and differs between the early and late phase. The association between *IL17A* gene polymorphisms and acute rejection (AR) of kidney allografts is well known [10, 11], and the use of IL-17 concentrations as an early marker of AR has been considered [12]. However, there are only a few reports linking this cytokine with chronic rejection, and studies analysing kidney allograft histopathological changes are lacking [13]. Previous studies have demonstrated that the G allele of the rs2275913 *IL17A* gene polymorphism is associated with significantly impaired long-term kidney allograft function and that the GA genotype of the rs11465553 *IL17F* gene polymorphism is associated with a significantly higher risk of a return to dialysis after kidney transplantation [14]. Because of the association between analyzed polymorphisms and post-transplant immune response, we assume that these single nucleotide polymorphisms (SNPs) may affect morphological structure of transplanted kidney through presence of glomerulitis, tubulitis, arteritis, cell infilitration and fibrosis.

The aim of this study was to examine the association of rs2275913 *IL17A* and rs2397084, rs11465553 and rs763780 *IL17F* gene polymorphisms with histopathological changes in transplanted kidney biopsies such as: glomerulitis, tubulitis, arteritis, cell infilitration and fibrosis.

## Methods

The study enrolled 82 consecutive Caucasian patients after renal allograft transplantation (48 males and 34 females, mean age: 47.63 ± 12.96 years) in whom a kidney biopsy was performed between 2000 and 2006 because of impaired graft function, 183 kidney transplant recipients in whom no kidney biopsy was performed and 168 healthy controls. All biopsies were reviewed by a renal pathologist, and the Banff working classification criteria were used. The recipient’s age and gender, the delayed graft function (DGF; which was defined as the need for dialysis during the first 7 days after transplantation), the AR occurrence and the number of episodes diagnosed clinically and confirmed by biopsy, chronic allograft nephropathy and chronic allograft dysfunction (CAD) were recorded in each case. All kidney allograft recipients were treated with the calcineurin inhibitor (cyclosporine A or tacrolimus), mycophenolate mofetil and glucocorticosteroids. The protocols differed between the patients because of changes in immunosuppressive therapies during the last 15 years. Additional patient characteristics are presented in Table 1. Blood samples for genetic analysis were collected from all patients at the start of the study, and informed consent was obtained from all patients. The local ethics committee at the Pomeranian Medical University, Szczecin, Poland, approved the study protocol (BN 120/2000) and written consent was obtained form all patients.

**Table 1:** Clinical characteristics of studied patients

Age [years]	47.55 ± 12.96
Sex [M/F]	48/34
Time after transplantation [months]	31 ± 9
Delayed graft function	31 patients
Creatinine [mg/dl]	1.76 ± 0.58
Cyclosporine A [ng/ml]	150–200
Tacrolimus [ng/ml]	7–12

### Genotyping

Genomic DNA was extracted from leukocytes contained in 200 μL whole blood samples using a GeneMATRIX Quick Blood DNA Purification Kit (EURx, Poland). A DNA concentration was approximately 300 ng/μl. The DNA was standardised using a Nanodrop ND-1000 spectrophotometer, and concentrations were equalised to 20 ng/μl. This material was used as the matrix for amplification in a real-time polymerase chain reaction (RT-PCR).

The rs2397084 T > C (Glu126Gly), rs11465553 G > A (Val155Ile) and rs763780 T > C (His167Arg) polymorphisms within the *IL17F* gene and the rs2275913 A > G (− 197 A > G) polymorphism within the *IL17A* gene promoter were genotyped using TaqMan genotyping assays on a 7500 FAST Real-Time PCR System (Applied Biosystems, USA).

### Statistical analysis

The distribution of the genotypes and alleles in the studied sample was initially evaluated with the Hardy–Weinberg equilibrium to assess if any population deviations occurred. The frequency of the genotypes and alleles was subsequently evaluated using the Chi-squared test or Fisher’s exact test. The severity of histopathological changes in renal allografts was compared between genotype groups using the nonparametric Kruskal–Wallis test followed by the Mann–Whitney test, as their distributions were significantly different from normal. *P* < 0.05 without correction for multiple comparisons was considered statistically significant. The study had sufficient statistical power to detect with 80% probability differences in the severity of histopathological changes between genotype groups measured according to Banff classification on rank scale ranging from 0 to 3 (Grade 0 = 0, Grade 1 = 1, Grade 2 = 2, Grade 3 = 3) equal to: between 0.39 and 0.73 (depending on histopathological parameter) of degrees of Banff scale for rs2275913, 0.55–1.03 for rs2397084, 0.44–0.90 for rs11465553 and 0.67–1.39 for rs763780. In summary, for most of the polymorphisms and histopathological parameters our study could detect the difference of a parameter between the genotype groups equivalent to one degree of Banff classification. It means that if real average value of Banff scale for the first genotype group is “1″ corresponding “Grade 1″ and for the second genotype group it is “2″ corresponding to “Grade 2″, then our study has enough statistical power to claim at *p* < 0.05 that the histopathological changes are more severe in the second group.

## Results

The distribution of all studied polymorphisms was consistent with the Hardy–Weinberg equilibrium (*P* = 0.59; *P* = 0.51; *P* = 1.0 and *P* = 1.0, respectively) and is shown in Additional file 1: Tables S1 and S2. As shown in Additional file 1: Tables S1 and S2 there were no statistically significant differences in distribution of studied polymorphisms between kidney transplant recipients in whom kidney biopsy was performed vs. kidney transplant recipients in whom no kidney biopsy was performed and between kidney transplant recipients in whom kidney biopsy was performed vs. control group.

There was a significant association between the rs2275913 *IL17A* gene polymorphism and the grade of tubulitis, which was more severe among renal allograft recipients with the A allele compared to patients with the GG genotype (GG vs. AG + AA, *P* = 0.02). Arteriolar hyaline thickening was also significantly correlated with this polymorphism; however, it was more severe among patients with the G allele compared to recipients with the AA genotype (AA vs. AG + GG, *P* = 0.01). In addition, mesangial matrix increase was significantly enhanced in patients with the rs2275913 *IL17A* G allele compared to recipients with the AA genotype (AA vs. GG + AG, *P* = 0.04). There were no statistically significant associations between the rs2275913 *IL17A* gene polymorphism and other histopathological changes in kidney allograft biopsies (Table 2).

**Table 2:** The associations between histopathological changes (i, t, v, ah, ci, cg, ct, cv, mm) in kidney allografts and rs2275913 *IL17A* gene polymorphism

Biopsy parameter and genotypes	Grade 0 *n* %		Grade 1 *n* %		Grade 2 *n* %		Grade 3 *n* %		Mean ± SD	M-W test p for comparison	
										GGvsAG+AA	AAvsAG+GG
(i) *n* = 72											
AA	2	20.00	4	40.00	2	20.00	2	20.00	1.40 ± 1.08		
AG	9	26.47	13	38.24	7	20.59	5	14.71	1.24 ± 1.02	0.96	0.68
GG	9	32.14	8	28.57	5	17.86	6	21.43	1.29 ± 1.15		
(t) *n* = 64											
AA	1	11.11	4	44.44	1	11.11	3	33.33	1.67 ± 1.12		
AG	5	17.24	13	44.83	9	31.03	2	6.90	1.28 ± 0.84	**0.02**	0.13
GG	11	42.31	10	38.46	3	11.54	2	7.69	0.85 ± 0.92		
(v) *n* = 57											
AA	6	85.71	1	14.29	0	0.00	0	0.00	0.14 ± 0.38		
AG	25	89.29	1	3.57	1	3.57	1	3.57	0.21 ± 0.69	0.37	0.64
GG	21	95.45	1	4.55	0	0.00	0	0.00	0.05 ± 0.21		
(ah) *n* = 68											
AA	6	66.67	3	33.33	0	0.00	0	0.00	0.33 ± 0.50		
AG	8	25.00	17	53.13	7	21.87	0	0.00	0.97 ± 0.70	0.45	**0.01**
GG	8	29.63	12	44.44	6	22.22	1	3.70	1.00 ± 0.83		
(ci) n = 68											
AA	6	60.00	4	40.00	0	0.00	0	0.00	0.40 ± 0.52		
AG	16	50.00	10	31.25	5	15.63	1	3.13	0.72 ± 0.85	0.61	0.32
GG	13	50.00	7	26.92	4	15.38	2	7.69	0.81 ± 0.98		
(cg) *n* = 55											
AA	7	100.0	0	0.00	–		0	0.00	0.00 ± 0.00		
AG	22	91.67	1	4.17	–		1	4.17	0.17 ± 0.64	0.14	0.29
GG	19	79.17	5	20.83	–		0	0.00	0.21 ± 0.42		
(ct) n = 68											
AA	3	30.00	6	60.00	0	0.00	1	10.00	0.90 ± 0.88		
AG	2	6.25	20	62.50	8	25.00	2	6.25	1.31 ± 0.69	0.97	0.11
GG	5	19.23	13	50.00	5	19.23	3	11.54	1.23 ± 0.91		
(cv) *n* = 51											
AA	6	75.00	1	12.50	0	00.00	1	12.50	0.50 ± 1.07		
AG	14	56.00	2	8.00	4	16.00	5	20.00	1.00 ± 1.26	0.79	0.38
GG	11	61.11	3	16.67	2	11.11	2	11.11	0.72 ± 1.07		
(mm) *n* = 60											
AA	7	87.50	1	12.50	0	0.00	0	0.00	0.13 ± 0.35		
AG	14	51.85	7	25.93	2	7.41	4	14.81	0.85 ± 1.10	0.39	0.04
GG	12	48.00	7	28.00	3	12.00	3	12.00	0.88 ± 1.05		

*p* value calculated with the Mann-Whitney’s (M-W) test

i – interstitial infiltration, t – tubulitis, v – intimal arteritis, ah – arteriolar hyaline thickening, ci – interstitial fibrosis, cg – glomerulopathy, ct – tubular atrophy, cv – fibrous intimal thickening, mm – mesangial matrix increase

As shown in Table 3, there were no significant associations between the rs2397084 *IL17F* gene polymorphism and histopathological changes in kidney allograft biopsies. Regarding the rs11465553 *IL17F* gene polymorphism, fibrous intimal thickening was significantly more severe among A allele carriers in comparison to patients with the GG genotype; however, these differences were on the border of statistical significance (GG vs. GA, *P* = 0.086). There were no statistically significant associations between the rs11465553 *IL17F* gene polymorphism and other histopathological changes in kidney allograft biopsies (Table 4).

**Table 3:** The associations between histopathological changes (i, t, v, ah, ci, cg, ct, cv, mm) in kidney allografts and rs2397084 *IL17F* gene polymorphism

Biopsy parameter and genotypes	Grade 0 n %		Grade 1 n %		Grade 2 n %		Grade 3 n %		Mean ± SD	M-W test p for comparison TTvsTC+CC	
(i) n = 72											
TT	15	25.86	19	32.76	13	22.41	11	18.97	1.35 ± 1.07		
TC	4	30.77	6	46.15	1	7.69	2	15.38	1.08 ± 1.04	0.26	
CC	1	100.0	0	0.00	0	0.00	0	0.00	0.00 ± 0.00		
(t) n = 64											
TT	13	25.49	22	43.14	11	21.57	5	9.80	1.16 ± 0.92		
TC	3	25.00	5	41.67	2	16.67	2	16.67	1.25 ± 1.06	0.90	
CC	1	100.0	0	50.00	0	0.00	0	0.00	0.00 ± 0.00		
(v) n = 57											
TT	42	91.30	2	4. 35	1	2.17	1	2.17	0.15 ± 0.56		
TC	9	90.00	1	10.00	0	0.00	0	0.00	0.10 ± 0.32	0.98	
CC	1	100.0	0	0.00	0	0.00	0	0.00	0.00 ± 0.00		
(ah) n = 68											
TT	18	32.14	26	46.43	12	21.43	0	0.00	0.89 ± 0.73		
TC	4	36.36	6	54.55	1	9.09	0	0.00	0.73 ± 0.65	0.90	
CC	0	0.00	0	0.00	0	0.00	1	100.0	3.00 ± 0.00		
(ci) n = 68											
TT	30	52.63	17	29.82	7	12.28	3	5.26	0.70 ± 0.89		
TC	5	50.00	4	40.00	1	10.00	0	0.00	0.60 ± 0.70	0.77	
CC	0	0.00	0	0.00	1	100.0	0	0.00	2.00 ± 0.00		
(cg) n = 55											
TT	36	87.70	4	9.76	–		1	2.44	0.17 ± 0.54		
TC	12	92.31	1	7.69	–		0	0.00	0.08 ± 0.28	0.88	
CC	0	0.00	1	100.0	–		0	0.00	1.00 ± 0.00		
(ct) n = 68											
TT	7	12.50	33	58.93	10	17.86	6	10.71	1.27 ± 0.82		
TC	3	27.27	6	54.55	2	18.18	0	0.00	0.91 ± 0.70	0.38	
CC	0	0.00	0	0.00	1	100.0	0	0.00	2.00 ± 0.00		
(cv) n = 51											
TT	23	57.50	5	12.50	6	15.00	6	15.00	0.88 ± 1.16		
TC	7	70.00	1	10.00	0	0.00	2	20.00	0.70 ± 1.25	0.47	
CC	1	100.0	0	0.00	0	0.00	0	0.00	0.00 ± 0.00		
(mm) n = 60											
TT	24	52.17	13	28.26	4	8.70	5	10.87	0.78 ± 1.01		
TC	9	69.23	2	15.38	1	7.69	1	7.69	0.54 ± 0.97	0.61	
CC	0	0.00	0	0.00	0	0.00	1	100.0	3.00 ± 0.00		

*p* value calculated with the Mann-Whitney’s (M-W) test

*i* interstitial infiltration, *t* tubulitis, *v* intimal arteritis, *ah* arteriolar hyaline thickening, ci – interstitial fibrosis, cg – glomerulopathy, ct – tubular atrophy, cv – fibrous intimal thickening, mm – mesangial matrix increase

**Table 4:** The associations between histopathological changes (i, t, v, ah, ci, cg, ct, cv, mm) in kidney allografts and rs11465553 *IL17F* gene polymorphism

Biopsy parameter and genotypes	Grade 0 *n* %		Grade 1 *n* %		Grade 2 *n* %		Grade 3 *n* %		Mean ± SD	M-W test p for comparison GGvsGA	
(i) *n* = 72											
GG	17	27.42	22	35.48	13	20.97	10	16.13	1.26 ± 1.04	0.79	
GA	3	30.00	3	30.00	1	10.00	3	30.00	1.40 ± 1.27		
(t) *n* = 64											
GG	15	27.27	24	43.64	10	18.18	6	10.91	1.13 ± 0.94	0.51	
GA	2	22.22	3	33.33	3	33.33	1	11.11	1.33 ± 1.00		
(v) *n* = 57											
GG	44	93.62	2	4.26	0	0.00	1	2.13	0.11 ± 0.48	0.18	
GA	8	80.0	1	10.00	1	10.00	0	0.00	0.30 ± 0.68		
(ah) *n* = 68											
GG	20	33.90	27	45.76	11	18.64	1	1.69	0.88 ± 0.77	0.60	
GA	2	22.22	5	55.56	2	22.22	0	0.00	1.00 ± 0.71		
(ci) *n* = 68											
GG	28	48.28	19	32.76	8	13.79	3	5.17	0.76 ± 0.89	0.21	
GA	7	70.00	2	20.00	1	10.00	0	0.00	0.40 ± 0.70		
(cg) *n* = 55											
GG	40	85.11	6	12.77	–		1	2.13	0.19 ± 0.54	0.26	
GA	8	100.0	0	0.00	–		0	0.00	0.00 ± 0.00		
(ct) *n* = 68											
GG	9	15.25	32	54.24	13	22.03	5	8.47	1.24 ± 0.82	0.56	
GA	1	11.11	7	77.78	0	0.00	1	11.11	1.11 ± 0.78		
(cv) *n* = 51											
GG	29	64.44	6	13.33	4	18.89	6	13.33	0.71 ± 1.10	0.086	
GA	2	33.33	0	0.00	2	33.33	2	33.33	1.67 ± 1.37		
(mm) *n* = 60											
GG	28	52.83	14	26.42	4	7.55	7	13.21	0.81 ± 1.06	0.36	
GA	5	71.43	1	14.29	1	14.29	0	0.00	0.43 ± 0.79		

*p* value calculated with the Mann-Whitney’s (M-W) test

*i* interstitial infiltration, *t* tubulitis, *v* intimal arteritis, *ah* arteriolar hyaline thickening, *ci* interstitial fibrosis, *cg* glomerulopathy, *ct* tubular atrophy, *cv* fibrous intimal thickening, *mm* mesangial matrix increase

Table 5 shows a significant association between the rs763780 *IL17F* gene polymorphism and interstitial fibrosis, which was more severe among individuals with the C allele than those with the TT genotype (TT vs. TC, *P* = 0.017). Similarly, tubular atrophy was enhanced in individuals with the C allele than those with the TT genotype; however, these differences were on the border of statistical significance (TT vs. TC, *P* = 0.09). There were no statistically significant associations between the rs763780 *IL17F* gene polymorphism and other histopathological changes in kidney allograft biopsies.

**Table 5:** The associations between histopathological changes (i, t, v, ah, ci, cg, ct, cv, mm) in kidney allografts and rs763780 *IL17F* gene polymorphism

Biopsy parameter and genotypes	Grade 0 *n* %		Grade 1 *n* %		Grade 2 *n* %		Grade 3 *n* %		Mean ± SD	M-W test p for comparison TTvsTC	
(i) *n* = 72											
TT	20	29.85	22	32.84	13	19.40	12	17.91	1.25 ± 1.08	0.43	
TC	0	0.00	3	60.00	1	20.00	1	20.00	1.60 ± 0.89		
(t) *n* = 64											
TT	17	27.87	24	39.34	13	21.31	7	11.48	1.16 ± 0.97	0.89	
TC	0	0.00	3	100.0	0	0.00	0	0.00	1.00 ± 0.00		
(v) *n* = 57											
TT	47	90.38	3	5.77	1	1.92	1	1.92	0.15 ± 0.54	0.49	
TC	5	100.0	0	0.00	0	0.00	0	0.00	0.00 ± 0.00		
(ah) *n* = 68											
TT	21	33.33	30	47.62	11	17.46	1	1.59	0.87 ± 0.75	0.34	
TC	1	20.00	2	40.00	2	40.00	0	0.00	1.20 ± 0.84		
(ci) *n* = 68											
TT	35	55.56	18	28.57	8	12.70	2	3.17	0.64 ± 0.83	0.017	
TC	0	0.00	3	60.00	1	20.00	1	20.00	1.60 ± 0.89		
(cg) *n* = 55											
TT	44	88.00	5	10.00	–		1	2.00	0.16 ± 0.51	0.65	
TC	4	80.00	1	20.00	–		0	0.00	0.20 ± 0.45		
(ct) *n* = 68											
TT	10	15.87	37	58.73	11	17.46	5	7.94	1.18 ± 0.79	0.09	
TC	0	0.00	2	40.00	2	40.00	1	20.00	1.80 ± 0.84		
(cv) *n* = 51											
TT	28	60.42	6	12.50	6	12.50	7	14.58	0.83 ± 1.15	0.75	
TC	3	75.00	0	0.00	0	0.00	1	25.00	0.75 ± 1.50		
(mm) *n* = 60											
TT	30	54.55	15	27.27	5	9.09	5	9.09	0.73 ± 0.97	0.72	
TC	3	60.00	0	0.00	0	0.00	2	40.00	1.20 ± 1.64		

p value calculated with the Mann-Whitney’s (M-W) test

i – interstitial infiltration, t – tubulitis, v – intimal arteritis, ah – arteriolar hyaline thickening, ci – interstitial fibrosis, cg – glomerulopathy, ct – tubular atrophy, cv – fibrous intimal thickening, mm – mesangial matrix increase

## Discussion

Based on the results of previous studies, it was hypothesised that functional polymorphisms within IL-17 genes may be associated with histopathological changes in transplanted kidney biopsies. The results of this study demonstrated that the rs2275913 *IL17A* gene polymorphism was significantly associated with tubulitis, arteriolar hyaline thickening and mesangial matrix increase and that the rs763780 *IL17F* gene polymorphism was correlated with the severity of tubular atrophy and interstitial fibrosis. However, it should be taken into account that the statistical significance was achieved without correction for multiple testing, and no significant association would remain significant after such correction. Our statistical power analysis assumed no such correction, and the numbers of patients needed to achieve the assumed power with the correction would greatly exceed logistic possibilities of our transplantation center. Therefore, these results from our exploratory study should be verified by further research.

The main reason of performing the biopsy in each case was deteriorating kidney function expressed through impaired eGFR (increase of creatinine concentration of at least 25%) accompanied by other clinical symptoms. The majority of biopsy specimens were collected during the first year after transplantation because of acute rejection (AR) suspicion. In 19 cases biopsy confirmed CAD and was performed between the first and the fifth year after transplantation. Thus, taking into consideration that kidney function deterioration resulted from AR or chronic allograft dysfunction (CAD) a typical treatment was used. The treatment outcome presented as follow-up observation has been already published [14]. The main conclusion of that paper was that the GG genotype of the rs2275913 IL17A gene polymorphism was associated with significantly impaired long-term kidney allograft function, whereas the GA genotype of the rs11465553 IL17F gene polymorphism with a significantly higher risk of graft function loss and return to dialysis after kidney transplantation [14].

A biopsy is the gold standard for diagnosing kidney allograft disorders as it allows the verification of the probable causes of organ function deterioration. Therefore biopsies are performed in kidney recipients with DGF or suspected AR to assess the extent of chronic rejection [15]. The Banff working classification criteria are currently used to assess renal transplant pathology and facilitate the selection of appropriate treatments [16]. Histopathological changes in transplanted kidneys are associated with many immunological and nonimmunological factors; for example, ischemia-reperfusion injury not only damages the organ in the early posttransplant period but also triggers chronic inflammation leading to progressive interstitial fibrosis [17]. Glomerulopathy, tubular atrophy and interstitial fibrosis are the main morphological causes of CAD.

Interleukins are key mediators of the alloresponse, and the role of IL-17 in kidney transplantation has yet to be elucidated [18, 19]. IL-17 is present in different types of rejection, as it can affect both the innate and acquired immune response through neutrophil activation and by acting on antigen-presenting cells. One important function of IL-17 is inducing the production of cytokines and chemokines, including IL-1β, IL-6, IL-8 and tumour necrosis factor-α (TNF-α) [20], therefore there is growing interest in the various functions of IL-17 in transplantation research. Loverre et al. showed that transplanted kidneys undergoing DGF and AR were characterised by significantly higher levels of IL-17(+) cells. Moreover, the number of IL-17(+) cells was significantly increased in grafts with T-cell mediated AR in comparison to grafts with morphological symptoms of antibody-mediated rejection, interstitial fibrosis, tubular atrophy and acute tubular damage due to calcineurin-inhibitor toxicity. Nevertheless, IL-17(+) cells were observed in all studied allografts [21, 22]. Previous studies showed that the G allele of the rs2275913 *IL17A* gene polymorphism was significantly correlated with a higher risk of DGF and higher long-term creatinine concentrations [14]. DGF occurs as a result of ischemia-reperfusion injury, and the outcomes of these complications include chronic lesions of the allograft which are manifested through tubular atrophy, interstitial fibrosis and mesangial matrix increase. Besides the histopathological changes, a decrease in allograft function can also be observed [23]. In this study, the G allele of the rs2275913 *IL17A* gene polymorphism was associated with a significantly enhanced mesangial matrix increase. This result is assumed to be a logical causal link with the earlier findings. Similarly, the C allele of the rs763780 *IL17F* gene polymorphism was correlated with higher long-term creatinine concentrations in previous studies, and this study reported its association with the severity of tubular atrophy and interstitial fibrosis.

Research regarding hepatitis B virus infection in kidney transplant and bone marrow recipients revealed that the A allele of the rs2275913 *IL17A* gene polymorphism was implicated in AR and graft-versus-host disease [24, 25]. In this study, the A allele of this polymorphism was significantly associated with tubulitis, which is the hallmark manifestation of the acute immune response. Arteriolar hyaline thickening, which is a type of arteriolosclerosis, is another histopathological lesion that is associated with ageing, hypertension, diabetes mellitus and, in the case of kidney transplant recipients, calcineurin inhibitor toxicity. Arteriolar hyalinosis is often found in kidney pathology because of its multifactorial background [26]. In this study, the severity of arteriolar hyaline thickening may be related to the mesangial matrix increase due to the association of these lesions with the G allele of the rs2275913 *IL17A* gene polymorphism; however, it may also be an incidental or calcineurin inhibitor toxicity-related result that requires further research.

Animal studies revealed that IL-17 was implicated in chronic rejection. A decreased immune response leading to prolonged allograft survival after heart transplantation was observed in IL-17 deficient mice [27]. Moreover, it was shown that Tregs were responsible for downregulation of Th17 and thus lowering IL-17 levels during chronic inflammation [28]. Recent studies in a murine model of kidney allograft confirmed that IL-17 deficiency or neutralisation was protective against rejection and, more importantly, that this process may be mediated by Tregs [29]. Unfortunately, studies regarding the role of IL-17 gene polymorphisms in chronic rejection in humans are lacking, and this makes comparing the obtained results rather difficult.

## Conclusions

The results of this study may suggest a possible association between the rs2275913 *IL17A* and rs2275913 *IL17A* gene polymorphisms and some histopathological changes in transplanted kidney biopsies. However, further studies are needed to get more insight into the functional relevance of the polymorphisms examined with respect to long term kidney allograft outcome.

## Additional file

Additional file 1: **Table S1.** The distribution of *IL17A* and *IL17F* genotypes in control group vs. kidney transplant recipients in whom kidney biopsy was performed. **Table S2.** The distribution of *IL17A* and *IL17F* genotypes in kidney transplant recipients in whom kidney biopsy was performed vs. kidney transplant recipients in whom no kidney biopsy was performed. (DOCX 22 kb)
