# Supplementary material for: IL17A and IL17F genes polymorphisms are associated with histopathological changes in transplanted kidney
Source: BMC Nephrol. 2019 Apr 8;20:124. doi: 10.1186/s12882-019-1308-z (PMC6454731; doi:10.1186/s12882-019-1308-z)
Supplement: Supplementary file 1 — Table S1. The distribution of IL17A and IL17F genotypes in control group vs. kidney transplant recipients in whom kidney biopsy was performed. Table S2. The distribution of IL17A and IL17F genotypes in kidney transplant recipients in whom kidney biopsy was performed vs. kidney transplant recipients in whom no kidney biopsy was performed. (DOCX 22 kb) [file 12882_2019_1308_MOESM1_ESM.docx]

Additional file 1:Table S1. The distribution of *IL17A* and *IL17F* genotypes in control group vs. kidney transplant recipients in whom kidney biopsy was performed

|  | **Control group** | | | **KTx recipients with a biopsy** | | |  | |
| --- | --- | --- | --- | --- | --- | --- | --- | --- |
|  | **n** | **%** | | **n** | **%** | | **p** | |
| ***IL17A* rs2275913** **genotype** | 168 | 100.0 | | 75 | 100.0 | |  | |
| GG | 59 | 35.1 | | 30 | 40.0 | |  | |
| AG | 84 | 50.0 | | 35 | 46.7 | | 0.76 | |
| AA | 25 | 14.9 | | 10 | 13.3 | |  | |
|  |  |  | |  |  | |  | |
| ***IL17A* rs2275913** **allele** |  |  | |  |  | |  | |
| G | 202 | 60.1 | | 95 | 63.3 | |  | |
| A | 134 | 39.9 | | 55 | 36.7 | | 0.5 | |
|  |  |  | |  |  | |  | |
| ***IL17F* rs763780** **genotype** |  |  |  | |  |  | |  |
| TT | 158 | 94.0 | 70 | | 93.3 |  | |  |
| CT | 9 | 5.4 | 5 | | 6.7 | 0.74 | |  |
| CC | 1 | 0.6 | 0 | | 0.0 |  | |  |
|  |  |  |  | |  |  | |  |
| ***IL17F* rs763780** **allele** |  |  |  | |  |  | |  |
| T | 325 | 96.7 | 145 | | 96.7 |  | |  |
| C | 11 | 3.3 | 5 | | 3.3 | 0.97 | |  |
|  |  |  |  | |  |  | |  |
| ***IL17F* rs11465553** **genotype** |  |  |  | |  |  | |  |
| GG | 151 | 89.9 | 65 | | 86.7 |  | |  |
| AG | 17 | 10.1 | 10 | | 13.3 | 0.51 | |  |
| AA | 0 | 0.0 | 0 | | 0.0 |  | |  |
|  |  |  |  | |  |  | |  |
| ***IL17F* rs11465553** **allele** |  |  |  | |  |  | |  |
| G | 319 | 94.9 | 140 | | 93.3 |  | |  |
| A | 17 | 5.1 | 10 | | 6.7 | 0.47 | |  |
|  |  |  |  | |  |  | |  |
| ***IL17F* rs2397084** **genotype** |  |  |  | |  |  | |  |
| TT | 132 | 78.6 | 60 | | 80.0 |  | |  |
| CT | 32 | 19.0 | 14 | | 18.7 | 0.86 | |  |
| CC | 4 | 2.4 | 1 | | 1.3 |  | |  |
|  |  |  |  | |  |  | |  |
| ***IL17F* rs2397084** **allele** |  |  |  | |  |  | |  |
| T | 296 | 88.1 | 134 | | 89.3 |  | |  |
| C | 40 | 11.9 | 16 | | 10.7 | 0.69 | |  |
|  |  |  |  | |  |  | |  |
|  |  |  |  | |  |  | |  |

p value calculated with χ^2^ test or Fisher’s exact test

Additional file 1: Table S2. The distribution of *IL17A* and *IL17F* genotypes in kidney transplant recipients in whom kidney biopsy was performed vs. kidney transplant recipients in whom no kidney biopsy was performed

|  | **KTx recipients without a biopsy** | | | **KTx recipients with a biopsy** | | |  | |
| --- | --- | --- | --- | --- | --- | --- | --- | --- |
|  | **n** | **%** | | **n** | **%** | | **p** | |
| ***IL17A* rs2275913** **genotype** | 183 | 100.0 | | 75 | 100.0 | |  | |
| GG | 77 | 42.1 | | 30 | 40.0 | |  | |
| AG | 88 | 48.1 | | 35 | 46.7 | | 0.71 | |
| AA | 18 | 9.8 | | 10 | 13.3 | |  | |
|  |  |  | |  |  | |  | |
| ***IL17A* rs2275913** **allele** |  |  | |  |  | |  | |
| G | 242 | 66.1 | | 95 | 63.3 | |  | |
| A | 124 | 33.9 | | 55 | 36.7 | | 0.55 | |
|  |  |  | |  |  | |  | |
| ***IL17F* rs763780** **genotype** |  |  |  | |  |  | |  |
| TT | 172 | 94.0 | 70 | | 93.3 |  | |  |
| CT | 11 | 6.0 | 5 | | 6.7 | 0.78 | |  |
| CC | 0 | 0.0 | 0 | | 0.0 |  | |  |
|  |  |  |  | |  |  | |  |
| ***IL17F* rs763780** **allele** |  |  |  | |  |  | |  |
| T | 355 | 97.0 | 145 | | 96.7 |  | |  |
| C | 11 | 3.0 | 5 | | 3.3 | 0.85 | |  |
|  |  |  |  | |  |  | |  |
| ***IL17F* rs11465553** **genotype** |  |  |  | |  |  | |  |
| GG | 168 | 91.8 | 65 | | 86.7 |  | |  |
| AG | 15 | 8.2 | 10 | | 13.3 | 0.25 | |  |
| AA | 0 | 0.0 | 0 | | 0.0 |  | |  |
|  |  |  |  | |  |  | |  |
| ***IL17F* rs11465553** **allele** |  |  |  | |  |  | |  |
| G | 351 | 95.9 | 140 | | 93.3 |  | |  |
| A | 15 | 4.1 | 10 | | 6.7 | 0.22 | |  |
|  |  |  |  | |  |  | |  |
| ***IL17F* rs2397084** **genotype** |  |  |  | |  |  | |  |
| TT | 146 | 79.8 | 60 | | 80.0 |  | |  |
| CT | 35 | 19.1 | 14 | | 18.7 | 0.98 | |  |
| CC | 2 | 1.1 | 1 | | 1.3 |  | |  |
|  |  |  |  | |  |  | |  |
| ***IL17F* rs2397084** **allele** |  |  |  | |  |  | |  |
| T | 327 | 89.3 | 134 | | 89.3 |  | |  |
| C | 39 | 10.7 | 16 | | 10.7 | 1.0 | |  |
|  |  |  |  | |  |  | |  |

p value calculated with χ^2^ test or Fisher’s exact test
